# The Skin

**Published:** 1877-05-20

**Authors:** L. P. Yandell

**Affiliations:** Professor of Therapeutics and Clinical Medicine


### THE SKIN.

From a Clinical Lecture by L. P. YANDELL, JR., M. D., Professor of Therapeutics and Clinical Medicine.

GENTLEMEN—The *vesiculæ* come next in the order adopted in the beginning of this course. A vesicle literally means a little bladder, and the vesicular skin diseases are those in which we find small elevations of the scarf-skin containing the watery portions of the blood, and constituting, in common parlance, little blebs or blisters. Under this head I shall describe the two forms of *miliaria* and the several varieties of *herpes*.

*Miliaria* (from *milium*, the millet seed) we find on the skins of persons confined to bed by febrile affections and especially where an excess of bed-clothing covers the patient. This is denominated *miliaria clinica*, or *sudamina alba*. The vesicles, from a pin's point to a pin's head in size, consist of tiny elevations of the epidermis, filled with translucent or transparent fluid, probably confined perspiration. No medication is called for.

*Miliaria rubra* is a more important malady. It is true dermatitis—that is an inflammation of the skin. The suffix *rubra* (meaning red) is given it because of its color.

You are all familiar with *miliaria rubra* under the title of "prickly heat." Minute vesicles crowning small, pointed, red elevations, crowded together in flocks covering large extents of cuticle, most usual on the arms and neck, but found on all portions of the body, and occasionally covering the entire cuticle and characterized by burning, stinging, pricking sensations—such is *miliaria rubra*.

Where the eruption is aggravated by irritants, and when the blood is in a depraved condition, prickly heat may degenerate or augment into eczema, pustules, furuncles. This is a disease of hot weather, and is peculiar to no period of life or class in society. Negroes have it less than whites. Its exciting causes are excessive heat, either solar or artificial; wearing flannel, too frequent bathing, the use of strong soap; in children urinary and fecal discharges allowed to remain in contact with the skin. Its predisposing causes are any conditions depressing vitality. *Malaria* is by far the most frequent predisposing cause. Dyspepsia, diarrhea, dentition, and the use of alcoholic stimulants are common predisposing causes. Its treatment is simple. Proper clothing, proper bathing, proper food and drink, should first be secured. Next attend to functional disturbances. Apply to the skin astringent and anodine ointments, if the skin be tolerably dry; if it be decidedly moist, use astringent and anodyne powders, such as chalk or bismuth combined with tannin and morphia. Bathing in solutions of salt or soda sometimes cures the eruption. *Quinine* in anti-periodic doses is our best remedy in severe cases, and seldom fails to cure. Soda should be given when acidity of the skin or digestive organs is manifest. Iron and the bitters are often demanded, and obstinate and chronic cases yield to arsenic. Mercurial cathartics in children are frequently beneficial.

*Herpes* we next take up. The name is from a Greek word, meaning to creep. The following are its chief varieties: *Herpes zoster* or *zona*, *H. phlyctenodes*, *H. febrilis*, *H. circinatus*, *H. preputialis*, *H. capitis*, *H. generalis*, *H. pudendalis*, *H. nasalis*, *H. auris*, *H. labilis*.

Herpes zoster, called by English-speaking people " Shingles," and by the Germans "fire-girdle," is quite rare. It consists in an incomplete zone or girth occurring on the trunk between the axillæ and the umbilicus, composed of vesicles from the size of a hemp seed to that of a bean. The eruption begins as a red papule. The vesicles are in clusters or flocks, forming a band a few inches to a foot in width. The vesicles are transparent, translucent, or straw-colored. They are situated on an angry red base. In some instances the two ends of the zone are on a level, and in others one may be many inches higher than the other. The popular belief is that if the eruption ever meets around the body, death is certain; and the popular and most ancient remedy is the blood of a black cat's tail applied to the eruption. Both ideas are unfounded in fact. The vesicles may dry into a crust and disappear in six to twenty days, or ulceration with profuse suppuration may take place, or an abundant and prolonged watery discharge may happen and the disease be indefinitely prolonged. Pain of two sorts may exist in connection with *herpes zoster*. It may be in the girdle, burning like fire, or stinging, or aching; or the pain may be only in the bones, and of a boring, aching character. The first usually ceases with the skin manifestation; the second may endure long after the eruption is gone. The etiology of this is often utterly obscure. It may be malarial, catarrhal, or rheumatic in its origin, and sometimes seems due to disease in the nerve centres. Many cases will disappear without treatment; most yield satisfactorily to proper remedies. Some are incurable. The indications for cure are to remove the cause, if this be practicable, and to relieve sypmtoms. Antiperiodics, tonics, anodyne, alteratives, are our most potent remedies.

I have encountered but one incurable case. The patient was a Prussian, seventy-six years old, in affluent circumstances, of splendid physique, and an enormous consumer of brandy, though never drunk. This was the first sickness of his life. The inflammatory pains in the skin and the neuralgic pains in the limbs, both were present in their most excruciating form. His case lasted nearly four months and was terminated by death from exhaustion. He got quinine, arsenic, iron, carbolic acid, the bisulphites, the iodides and bromides, mercury, the alkalies, cod-liver oil, sulphur, purgatives, strychnia and aconite without benefit. All the changes of hypodermic medication were rung on him with the result of only temporary relief. He took the various forms of opium, belladonna, Indian hemp, chloral and alcohol, etc. His pain seemed enhanced in intensity from week to week, and he declared he suffered the tortures of the damned. During the latter portion of his life he was kept under the influence of alcohol or opium, which he consumed in immense quantities. These two agents gave him the most certain and most prolonged oblivion to pain. You have before you a perfect representation of all the varieties of herpes in the plates and models, which are correctly labeled. On the trunk you perceive the band of vesicles constituting zoster, and on the forehead the patch of vesicles called *herpes phlyctenodes.* You observe this herpes appears in isolated clusters and not in a band or zone. It is less formidable than zoster. Its etiology and treatment are that of "fire-girdle." *Herpes febrilis*, or fever-blisters, includes all the other herpes. They derive their names from their location or shape. When first formed these herpetic vesicles are observed to be divided into cells. They may dry up, suppurate, or remain long *in statu quo.* They may go and come in successive crops. They are of malarial origin most frequently. They are not infrequently of catarrhal origin—that is, from cold. Dyspepsia, dentition, and uterine derangements may give rise to these herpes; so may a decayed tooth.

*Herpes labialis* (fever blisters on the mouth) occasionally in this climate, in bad cases, and more frequently further South, swells the lip to three or four, or even ten times its normal size, and gives rise to exquisite pain. These blisters are first white and then yellow.

*H. capitis*, by matting the hair of the head and accumulating large crusts, is often vexatious to its possessor.

*H. circinatus* and *H. iris* are varieties of the same herpes, presenting rings of little blisters. It is especially a disease of the aged. It is sometimes mistaken for ringworm.

*H. preputialis* and *pudendalis*, unless aggravated by acid discharges or harsh treatment, are in themselves most insignificant affairs. But, because of their location, they become, indirectly, most serious sores. *They are the doors by which syphilis most often enters the system.* Unbroken skin and sound mucus membrane are a cuirass, indeed a perfect defensive armor against syphilis. It is only when these coats are penetrated by inflammatory destruction, or by an abrasion, that the dread malady is dangerous by direct contact.

The treatment of the several varieties of herpes just considered is both simple and satisfactory. Remembering the causes I have enumerated, you are to discover and remove these if possible. Locally apply tannin and morphine in powder, solution, or ointment, as may seem indicated in each particular case. Use no soap on the eruptions. *Above all things, never burn them.* By the by, this herpes is seldom seen in its veciscular stage. When brought to our notice we usually see small yellowish or grayish ulcers—the basis of the blisters.

Quinine is by all odds our best and most certain cure for these herpetic manifestations. Iron is almost always needed. Arsenic is well in the chronic cases. Soda should be given when acidity of stomach exists, and opium when catarrhal fever is the source of the herpes.—*Cincinnati Medical News.*

[We have found a solution of tannin in glycerine gr. x to the ounce a good local application in these affections.—Eds.]

